# Reference ranges and reproducibility studies for right heart myocardial deformation by feature tracking cardiovascular magnetic resonance imaging^☆^

**DOI:** 10.1016/j.dib.2017.11.037

**Published:** 2017-11-12

**Authors:** Boyang Liu, Ahmed M. Dardeer, William E. Moody, Nicola C. Edwards, Lucy E. Hudsmith, Richard P. Steeds

**Affiliations:** a University of Birmingham Institute of Cardiovascular Sciences, Birmingham, UK; b University Hospitals Birmingham NHS Foundation Trust, Birmingham, UK; c Minia University, Minia, Egypt

**Keywords:** CMR, cardiac magnetic resonance, LV, left ventricular, RV, right ventricular, EF, ejection fraction, EDVi, indexed end diastolic volume, ESVi, indexed end systolic volume, LVMi, indexed left ventricular mass, eGFR, estimated glomerular filtration rate, RV Ell, right ventricular peak longitudinal strain, SR_S__′_, peak systolic strain rate, SR_E__′_, peak early diastolic strain rate, SR_A__′_, peak late diastolic strain rate

## Abstract

Feature tracking of the right heart on cardiac MRI is a novel and promising technique for the measurement of right heart myocardial strain. We present here the reference ranges for right ventricular longitudinal strain and strain rate, as well as peak strain of the right atrium within a cohort of 100 healthy individuals recruited from the UK.

We present data on the reproducibility of these feature tracking techniques and explore relationship between strain and baseline demographic parameters.

**Specifications Table**

**Table:** 

Subject area	*Medicine*
More specific subject area	*Cardiac imaging*
Type of data	*Table, graph*
How data was acquired	*Feature tracking cardiac magnetic resonance (FT-CMR) was used to assess right atrial (RA) and right ventricular (RV) strain and strain rate using commercially available cvi42 software (version 5.3.4, Circle Vascular Imaging, Canada)*
Data format	*Analysed data presented*
Experimental factors	*This cohort of 100 healthy subjects was constructed to contain 10 males and 10 females from each decade of life between the ages of 20 and 70.*
Experimental features	*The endo- and epicardial boundaries of the RA and RV within anonymized CMR studies were defined by observer 1 using cvi42 software. To generate reference ranges, peak longitudinal strain and strain rates were obtained for the RV; peak longitudinal strain rate was obtained for the RA. Observer 1 repeated analyses following 1 months for intra-observer variability. Observer 2 performed blinded analyses for inter-observer variability assessment.*
Data source location	*University Hospital Birmingham NHS Foundation Trust, UK*
Data accessibility	*Summaries of data are presented within this article. Raw data can be supplied to readers upon reasonable request.*

**Value of the data**

• The quantification of right heart function in a reproducible and repeatable manner is vital for the monitoring of both congenital and acquire cardiac diseases.
• This process is normally time-consuming, difficult and require meticulous care even in the era of semi-automated boundary detection. Myocardial deformation is a novel and promising technique which may be able to overcome this challenge.
• Right heart strain imaging using feature tracking cardiac MRI does not require additional dedicated cardiac magnetic resonance (CMR) sequences such as tagging, but can instead be formed on routine cine studies, and is not limited by the availability of high quality echo windows as is the case for speckle tracking.
• We present here the reference ranges of RA and RV strain and strain rates within our healthy cohort of subjects. Clinically this data can be used to diagnose and monitor patients with reduced strain. We also welcome future research collaborations for which our cohort can act as age- and gender-matched controls of studies that are interested in the function of the right heart.
• Two datasets were produced with and exclusion of the septum to reflect different approaches to the contribution of the septum to RV function.

## 1 Data

There is increasing evidence for the prognostic value of right heart myocardial deformation (strain and strain rate) in the monitoring of both acquired and congenital heart diseases.

We present the reference ranges of right ventricular (RV) longitudinal strain (Ell) and strain rates obtained from feature tracking cardiac MRI (FT-CMR) according to deciles of age, as well as the results of intra- and inter-observer reproducibility studies on the measurement of these markers of right heart function. RV strain and strain rates were measured using two different techniques – that of RV free wall strain (FW Ell), and RV free wall plus septum (FW+S Ell). Right atrial peak strain reproducibility studies are also presented.

## 2 Experimental design, materials and methods

Full experimental design has been described elsewhere [1], but in brief, a cohort of 100 normal healthy subjects, containing 10 men and 10 women from each age decile between 20 and 70 years was constructed. Subjects were in optimal health and free from a history of hypertension, diabetes, obesity, dyslipidemia, or any cardiovascular, renal, hepatic, haematological and systemic inflammatory disease as assessed through clinical history and examination. All subjects had normal blood count and serum electrolytes. The QRISK2 score for each subject was calculated; this online calculator (www.qrisk.org) is widely used within the UK's National Health Service to predict an individual's risk of developing cardiovascular disease over the next 10 years [2].

CMR imaging was conducted using a 1.5-T scanner (Magnetom Avanto, Siemens, Germany). Right ventricular strain and strain rates were derived using commercially available Cvi42 software (version 5.3.4, Circle Vascular Imaging, Canada). Cvi42 utilizes an incompressible volume-based algorithm, which has been previously validated to produce accurate biventricular anatomical tracking [3]. From the horizontal long axis view, right ventricular 2D longitudinal (Ell) strain as well as strain rates (peak systolic strain rate SRS′; peak early diastolic strain rate SRE′; peak late diastolic strain rate SRA′) were defined in the region of interest between the endocardial and epicardial borders. RV Ell was defined as the peak point on the strain curve which is present at, or prior to end-systole. Endocardial and epicardial borders were drawn around the largest right atrial (RA) area to coincide with the RV end-systolic phase for RA Ell.

For the assessment of reproducibility, all CMR studies were anonymized prior to strain analysis. Observer 1 (AD) performed tissue tracking analysis for all 100 subjects, with a second analysis repeated in a randomly generated subset of 10 patients after a 1-month interval. For inter-observer variability, observer 2 (BL) independently feature tracked the randomly generated set of 10 scans.

### 2.1 Baseline demographics

**Fig. 1:**
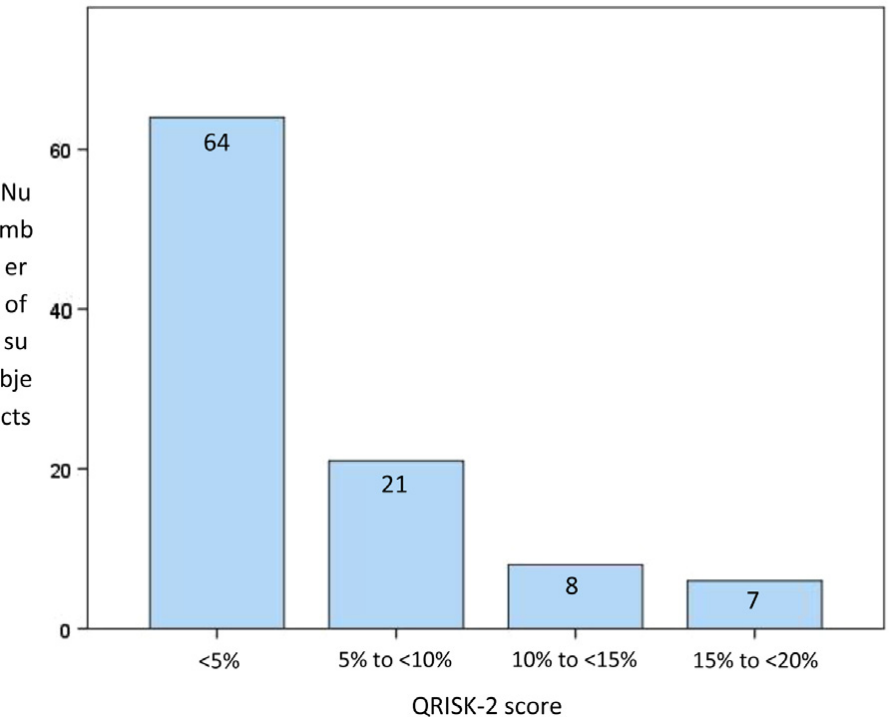
Graph illustrating QRISK-2 distribution of the 100-subject cohort.

The baseline demographics, ventricular volumes and function for the full cohort are listed in Table 1. All participants had a QRISK-2 score of <20% (Fig. 1).

**Table 1:** Baseline demographics of 100 healthy subjects.

	Female	Male	Overall	*P*
	(*n*=50)	(*n*=50)	(*n*=100)	
Age (years)	44.8±14.3	44.7±14.3	44.8±14.3	0.93
Height (cm)	163.8±5.6	178.2±8.6	171.2±10.2	0.03
Weight (kg)	69.9±11.7	80.9±12.8	75.5±13.4	0.73
BSA (m^2^)	1.8±0.2	2.0±0.2	1.9±0.2	0.78
LVEF (%)	70.5±6.7	70.8±6.7	70.7±6.7	0.79
LVEDVi (ml/m^2^)	64.1±13.1	65.5±11.6	64.8±12.3	0.97
LVESVi (ml/m^2^)	19.4±7.5	19.6±7.0	19.5±7.2	0.75
LVMi (kg/m^2^)	52.1±9.9	62.9±12.1	57.4±12.2	0.44
RVEF (%)	67.5±8.4	66.3±7.1	66.9±7.8	0.14
RVEDVi (ml/m^2^)	63.4±13.2	68.4±14.2	65.8±13.9	0.41
RVESVi (ml/m^2^)	21.0±8.0	23.7±9.5	22.3±8.8	0.51
Haemoglobin (g/L)	13.1±0.8	14.5±1.0	13.8±1.2	0.25
eGFR (mL/min)	85.1±13.5	88.8±12.7	86.8±13.2	0.89

Values are given as mean±standard deviation. *P* values represent independent *T*-test for male vs. female.

### 2.2 Right heart strain and strain rate

The peak RV Ell strain, RV strain rate values are presented in Table 2. As there were no consistent relationship between age and RV strain or strain rates on linear regression analysis, we have therefore provided values of the overall cohort. There were no gender differences for RV (FW-Ell *P*=0.32; FW+S Ell *P*=0.61) and RA strain (*P*=0.36) (Table 3).

**Table 2:** Reference values and regression analysis for RV strain, strain rate and RA strain according age.

	**Age deciles**					**Regression analysis**				**Overall cohort**
	≥20to<30	≥30to<40	≥40to<50	≥50to<60	≥60to<70	*R*	*R*^2^	*β*	*P*	
**FW+S Ell**	−21.3±0.22	−21.1±2.59	−22.6±3.43	−22.7±2.10	−21.6±4.37	0.10	0.01	−0.02	0.33	−*21.9*±*3.24*
**FW Ell**	−23.9±3.54	−23.2±3.42	−24.6±3.54	−25.4±2.94	−23.9±4.36	0.10	0.01	−0.02	0.35	−*24.2*±*3.59*
FW+S SR S′	−1.40±0.47	−1.48±0.41	−1.50±0.32	−1.42±0.35	−1.44±0.44	0.01	<0.01	<0.01	0.92	−*1.45*±*0.39*
FW+S SR E′	1.18±0.24	1.08±0.25	1.02±0.21	1.01±0.29	0.92±0.25	−0.30	0.09	−0.005	0.002	*1.04*±*0.26*
FW+S SR A′	0.87±0.24	0.86±0.31	0.89±0.29	1.05±0.36	1.00±0.40	0.24	0.054	0.005	0.018	*0.94*±*0.33*
FW SR S′	−1.48±0.34	−1.47±0.39	−1.55±0.39	−1.63±0.42	−1.59±0.49	0.14	0.02	−0.04	0.16	−*1.54*±*0.41*
FW SR E′	1.14±0.27	1.01±0.33	1.02±0.34	1.02±0.25	1.02±0.45	0.10	0.01	−0.002	0.31	*1.04*±*0.33*
FW SR A′	1.09±0.33	1.02±0.35	1.09±0.32	1.09±0.31	1.11±0.37	0.08	0.01	0.002	0.43	*1.08*±*0.33*
**RA Ell**	−22.1±3.72	−20.8±4.24	−21.0±3.39	−21.5±3.90	−20.1±3.59	0.14	0.02	0.04	0.18	−*21.1*±*3.76*

Strain values are presented in red; strain rate values are presented in black font.

**Table 3:** Reference values for RV strain, strain rate and RA strain according to gender.

	**Men**	**Women**	***P***
**FW+S Ell**	−21.6±3.36	−22.2±3.12	0.32
**FW Ell**	−23.9±3.59	−24.6±3.59	0.34
FW+S SR S′	−1.54±0.39	−1.35±0.38	0.017
FW+S SR E′	1.04±0.27	1.04±0.25	0.86
FW+S SR A′	0.96±0.37	0.92±0.29	0.54
FW SR S′	−1.62±0.46	−1.46±0.32	0.061
FW SR E′	1.06±0.35	1.01±0.32	0.42
FW SR A′	1.09±0.36	1.07±0.30	0.81
**RA Ell**	−20.6±4.10	−21.5±3.38	0.23

Strain values are presented in red; strain rate values are presented in black font. *P* values are derived from two-tailed independent samples *T*-test.

There was a weak correlation between height and RV FW+S Ell (*r*=0.21, *P*=0.05), but this was not present for RV FW-Ell, nor were there any significant correlations between RV strain and the parameters of weight, body mass index or body surface area.

### 2.3 Reproducibility studies

**Fig. 2:**
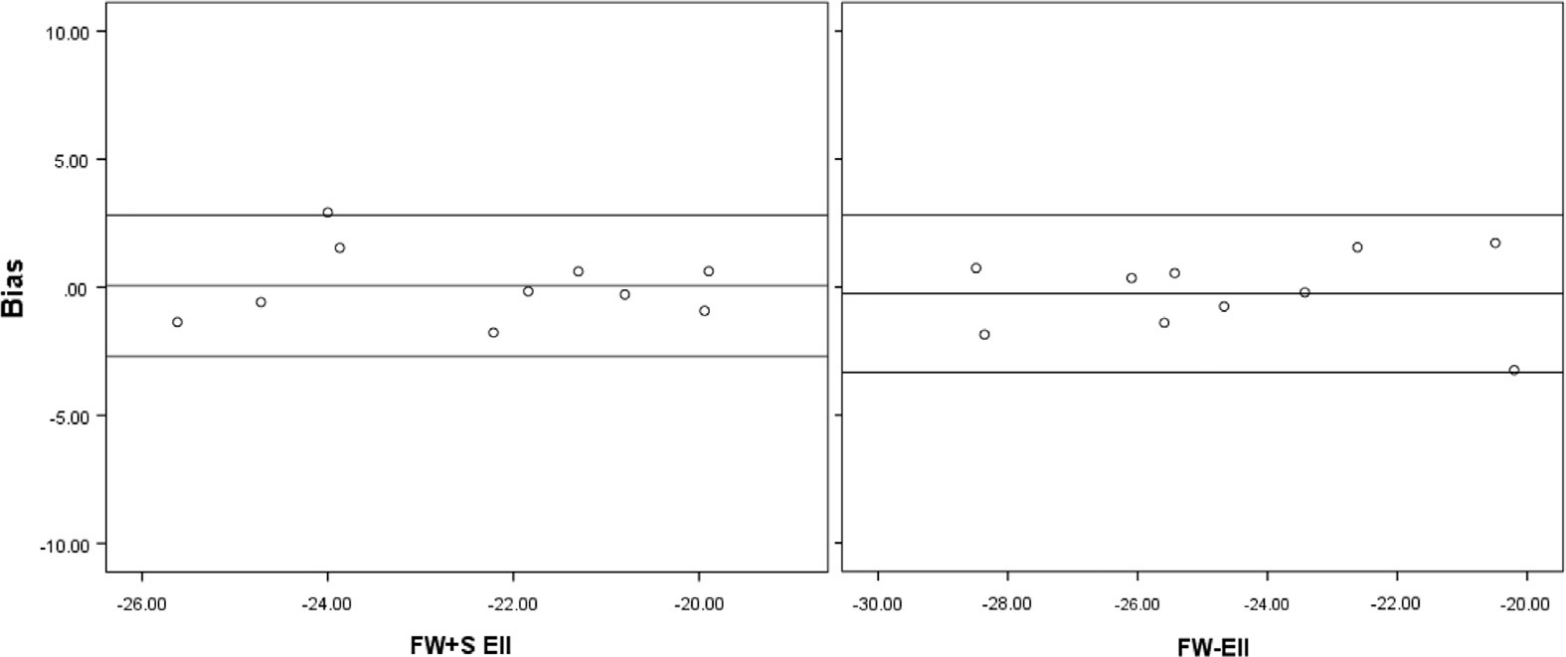
Bland-Altman plots illustrating inter-observer bias for RV Ell.

**Fig. 3:**
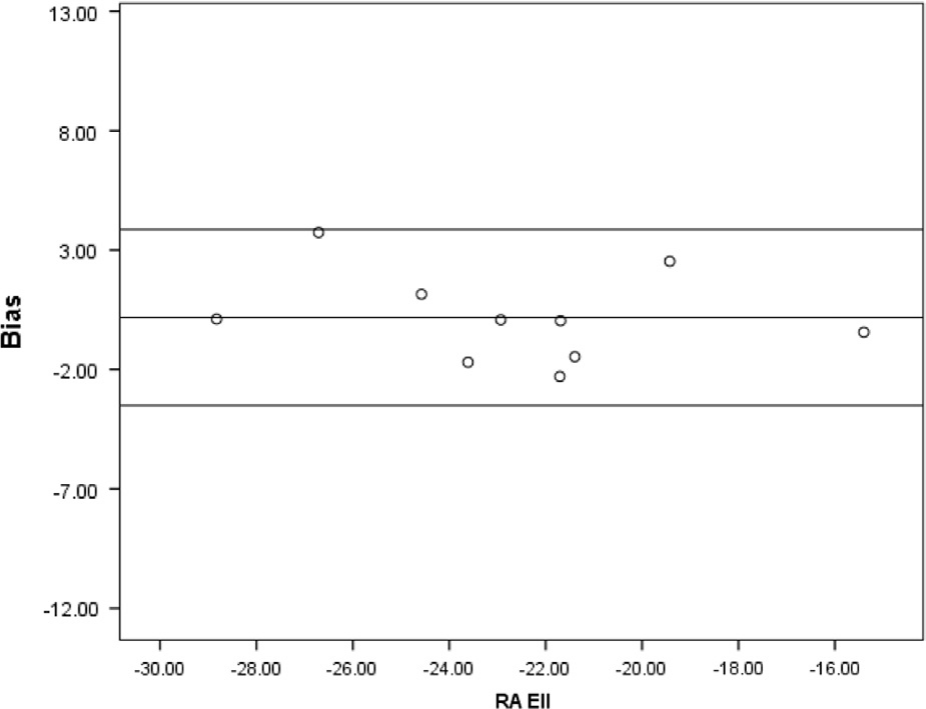
Bland-Altman plots illustrating inter-observer bias for RA Ell.

Intra- and inter-observer reproducibility studies are presented in Table 4 for RV FW+S and Table 5 for RV FW. RA reproducibility is presented in Table 6. The magnitude of biases are presented graphically on Bland Altman plots (Fig. 2, Fig. 3).

**Table 4:** Reproducibility studies for RV free-wall plus septum deformation.

	Variability	Mean bias±SD	Limits of agreement	ICC (95% CI)
RV FW+S Ell	*Intra-observer*	0.67±0.79	−1.27 to 1.81	0.92 (0.72–0.98)
	*Inter-observer*	1.08±1.41	−2.7 to 2.82	0.80 (0.54–0.97)
SR_S__′_	*Intra-observer*	0.16±0.13	−0.10 to 0.39	0.64 (−0.06 to 0.91)
	*Inter-observer*	0.21±0.27	−0.48 to 0.58	0.52 (−0.31 to 0.92)
SR_E__′_	*Intra-observer*	0.12±0.12	−0.28 to 0.19	0.92 (0.71–0.98)
	*Inter-observer*	0.17±0.18	−0.29 to 0.41	0.84 (0.67–0.98)
SR_A__′_	*Intra-observer*	0.16±0.22	−0.52 to 0.35	0.64 (0.1–0.89)
	*Inter-observer*	0.18±0.23	−0.58 to 0.32	0.53 (−0.2 to 0.85)

**Table 5:** Reproducibility studies for RV free-wall deformation.

	Variability	Mean bias±SD	Limits of agreement	ICC (95% CI)
RV free wall Ell	*Intra-observer*	1.08±0.97	−1.85 to 1.93	0.92 (0.61–0.98)
	*Inter-observer*	1.23±1.57	−2.71 to 2.19	0.87 (0.57–0.97)
SR_S__′_	*Intra-observer*	0.22±0.25	−0.37 to 0.61	0.28 (−0.28 to 0.74)
	*Inter-observer*	0.19±0.33	−0.56 to 0.69	0.38 (0.9–0.89)
SR_E′_	*Intra-observer*	0.17±0.24	−0.45 to 0.37	0.71 (0.18–0.92)
	*Inter-observer*	0.17 ±0.22	−0.38 to 0.49	0.69 (0.17–0.91)
SR_A__′_	*Intra-observer*	0.25±0.25	−0.68 to 0.30	0.64 (0.047–0.89)
	*Inter-observer*	0.25±0.29	−0.73 to 0.45	0. 64 (0.2–0.94)

**Table 6:** Reproducibility studies for RA longitudinal strain.

	**Variability**	**Mean bias±SD**	**Limits of agreement**	**ICC (95% CI)**
**RA E**_**ll**_	*Intra-observer*	1.68±1.86	−4.04 to 3.28	0.92 (0.72–0.98)
	*Inter-observer*	1.36±1.88	−3.52 to 3.87	0.89 (0.62–0.97)

## Transparency document Supplementary material

Supplementary material.
