# Supplementary material for: Reference ranges and reproducibility studies for right heart myocardial deformation by feature tracking cardiovascular magnetic resonance imaging
Source: Data Brief. 2017 Nov 12;16:244–9. doi: 10.1016/j.dib.2017.11.037 (PMC5713006; doi:10.1016/j.dib.2017.11.037)
Supplement: Supplementary file 1 — Supplementary material [file mmc1.docx]

**Conflict of Interest**

The authors declare no conflict of interest.
